# CXCR5+CD8+ T cells are localized in B cell follicles and germinal centers and exhibit regulatory and anti-tumor function

**DOI:** 10.1186/2051-1426-3-S2-P321

**Published:** 2015-11-04

**Authors:** Fuliang Chu, Sattva S Neelapu

**Affiliations:** 1 The University of Texas MD Anderson Cancer Center, Houston, TX, USA

## 

Germinal centers (GC) are specialized anatomic sites where B cells undergo somatic hypermutation, clonal expansion, and affinity-based selection. Stringent regulation of GC reactions is critical for homeostatic B cell development, as well as T-dependent humoral immunity against self and foreign antigens. Follicular helper T cells (Tfh, CXCR5^hi^PD^hi^Bcl-6^+^CD4^+^) that reside in GC serve as specialized B helper T cells and provide survival and selection signals to GC B cells. Despite recent understandings of Tfh function in autoimmunity, immunodeficiency, and B-cell lymphoma, mechanisms that regulate Tfh development and function remain limited.

In the current study, we determined the presence of CD45RA^-^CXCR5^+^CD8^+^ T cells in human peripheral blood, tonsillar tissues, and follicular lymphoma (FL) tumor samples by FACS and confocal microscopy. We found that CXCR5^+^CD8^+^ T cells were present in high numbers and localized to GCs and T cell zones in the tonsillar tissues and FL, but present in low numbers in the peripheral blood. Surface marker analysis suggested that these cells displayed an activated status. Importantly, FL tumor samples had significantly more CXCR5^+^CD8^+^ T cells than tonsillar tissues. Further analysis using intracellular cytokine staining showed that CXCR5^+^CD8^+^ T cells produced higher levels of IFN-γ and TNF-α compared to CXCR5^-^CD8^+^ subset.

Next, we performed T-B cell co-culture experiments to evaluate whether CXCR5^+^CD8^+^ T cells could regulate Tfh function in humans. FACS-sorted Tfh cells were co-cultured with naïve or memory B cells in the presence or absence of CXCR5^+^CD8^+^ T cells. These experiments indicated that CXCR5^+^CD8^+^ T cells suppressed Tfh function, as demonstrated by reduced differentiation of naïve and/or memory B cells into plasmablasts cells (CD19^int^CD38^+^). To test CXCR5^+^CD8^+^ T cell function in vivo, we employed the EG7 lymphoma model in TCR^-/-^ mice. Briefly, ovabulmin-primed OT1-specific CXCR5^+^CD8^+^ T cells were adoptively transferred into EG7 tumor-bearing TCR^-/-^ mice, and tumor size and survival rate were determined.

In summary, our data suggest that CXCR5^+^CD8^+^ T cells may have multiple roles, as they not only inhibited Tfh function, but also exhibited strong anti-tumor immunity.

